# Enhancing Chinese preschoolers’ executive function *via* mindfulness training: An fNIRS study

**DOI:** 10.3389/fnbeh.2022.961797

**Published:** 2022-08-25

**Authors:** Sha Xie, Chaohui Gong, Jiahao Lu, Hui Li, Dandan Wu, Xinli Chi, Chunqi Chang

**Affiliations:** ^1^Department of Early Childhood Education, Faculty of Education, Shenzhen University, Shenzhen, China; ^2^School of Biomedical Engineering, Health Science Center, Shenzhen University, Shenzhen, China; ^3^Shanghai Institute of Early Childhood Education, Shanghai Normal University, Shanghai, China; ^4^Macquarie School of Education, Macquarie University, Sydney, NSW, Australia; ^5^Department of Early Childhood Education, The Education University of Hong Kong, Hong Kong, Hong Kong SAR, China; ^6^School of Psychology, Shenzhen University, Shenzhen, China; ^7^Peng Cheng Laboratory, Shenzhen, China

**Keywords:** executive function, mindfulness training, fNIRS, preschooler, cognitive shifting, inhibitory control, working memory

## Abstract

Mindfulness training has been found to enable cognitive and emotional awareness and diminish emotional distraction and cognitive rigidity. However, the existing intervention studies have largely focused on school children, adolescents, and adults, leaving young children unexplored. This study examined the influence of mindfulness training on young children using the one-group pretest-posttest design. Altogether 31 Chinese preschoolers (*M*_*age*_ = 67.03 months, SD = 4.25) enrolled in a 5-week, twice-per-week mindfulness training. Their cognitive shifting, inhibitory control, and working memory were examined using a battery of executive function tasks. And their brain activations in the region of interest during the tasks were measured using fNIRS before and after the intervention. Results showed that their cognitive shifting and working memory tasks performance significantly improved, and their activation in the DLPFC significantly changed. Implications for this study were also included.

## Introduction

Executive function (EF) refers to the ability to control one’s actions and thoughts consciously, which is considered a higher mental process (Zelazo and Müller, 2010; Griffin et al., 2016) and conducive to children’s school readiness (Blair, 2002; Guedes et al., 2022). EF is developed in the early years to support children’s ability to regulate their behavior (Moriguchi and Hiraki, 2011) and, in turn, develop their later social, emotional, and cognitive competence (Griffin et al., 2016). Preliminary evidence has shown that mindfulness training considerably improves young children’s executive function and alleviates problem behaviors (Flook et al., 2015; Razza et al., 2015). However, most studies lack a comprehensive examination of EF’s three components, and very few have provided neuroimaging evidence to support the training effect. Thus, this study is dedicated to filling this research gap.

### Executive function and its three components

Miyake et al.’s (2000) influential work fractionate EF into three sub-domains that provide a foundation to regulate thoughts, behaviors, and emotions: (1) working memory, the ability to hold in mind information; (2) inhibitory control, the ability to inhibit fast and unthinking responses to stimulation; and (3) cognitive shifting, the ability to flexibly shift the focus of one’s mental frame (Blair, 2016; Moriguchi, 2017). It develops during infancy, shows important developmental changes in preschool years (Zelazo and Müller, 2010), and varies by task (Huizinga et al., 2006). The existing studies have provided evidence to support the relationship between emerging executive functions and maturation of the prefrontal cortex (Moriguchi, 2017; Smith et al., 2017), yet less is known about the relation between brain development and specific executive functions. A series of structural equation models indicated that each sub-domain of EF plays a differential role in performance on a range of executive outcome measures, highlighting the need to recognize the diversity of these sub-processes. Recently, fNIRS has been used to measure task-related changes in cerebral hemodynamics, measurable and doable for very young children. Accordingly, a range of executive function tasks has been explored in preschoolers using fNIRS, including working memory (Tsujii et al., 2009; Buss et al., 2014), inhibitory control (Inoue et al., 2012; Mehnert et al., 2013), and cognitive shifting (Moriguchi and Lertladaluck, 2019; Li et al., 2021b).

### Advances in studying executive function’s three components

First, the fNIRS studies on cognitive shifting, the ability to flexibly shift between tasks or mental states, have made noticeable progress. In particular, the Dimensional Change Card Sort (DCCS) task is widely used to measure 3 to 6-year-olds’ development of cognitive shifting (Zelazo, 2006). The existing fNIRS studies have found that 5-year-olds and adults could perfectly complete the task and show significant activation in the bilateral inferior prefrontal areas; only those 3-year-olds would perseverate to the previous rules (Moriguchi and Hiraki, 2011, 2014). Furthermore, 5-year-olds who are heavy users of tablets performed worse in the DCCS tasks and showed significantly different activations from non-users (Li et al., 2021a). The unexpected synchronous increase in HbO and HbR was similar to those during epileptic seizures (Pouliot et al., 2012). These findings jointly suggest that prefrontal cortex activations play an important role in successful shifting during the DCCS task and that individual differences might be associated with activation patterns.

Second, the fNIRS studies on inhibitory control, the ability to consciously inhibit a pre-potent response, have also attained some achievements. The go/no-go paradigm measures the response inhibition by requiring the subject to respond to a frequent target stimulus and suppressing the repress of the occurrence of a rare non-target stimulus (Mehnert et al., 2013). The existing fNIRS studies with the go/no-go task have revealed significant activation in both go and no-go trials in the right frontal and parietal regions. Furthermore, the functional connectivity analysis revealed that children ages 4–6 years showed stronger partial coherence in short-range connectivity in the right frontal and right parietal cortices than adults (Mehnert et al., 2013). These findings jointly suggest that right frontal and parietal activations might play an important role in performing the go/no-go task.

Third, the fNIRS studies on working memory, the ability to save, manipulate, and remember information, have also advanced in recent years. Several tasks have been designed to measure young children’s working memory and its neural basis using fNIRS. In general, significant activation in the frontal and parietal cortex has been found during the working memory task (Tsujimoto et al., 2004; Buss et al., 2014), and child age was positively related to the increase in lateral prefrontal cortex (LPFC) activation, accuracy, and response speed (Perlman et al., 2016). These findings indicated the age-related changes in the prefrontal function, providing empirical evidence to support the effective development of EF during early childhood. However, the above-mentioned studies only focused on one aspect of children’s EF and have seldomly examined all three components simultaneously, limiting our understanding of the development of the neural correlates of EF during early childhood, the critical period for the maturation of executive function.

### Advances in early intervention studies

Various approaches and interventions have been introduced to enhance young children’s EF, among which mindfulness practices have been widely implemented in early childhood classrooms (Razza et al., 2015; Li et al., 2019; Ren et al., 2019). The Intention-Attention-Attitude (IAA) model provides a mechanism of actions underlying mindfulness-based interventions (Shapiro et al., 2006). In this model, the potential mechanism of mindfulness is suggested as “intentionally (I) attending (A) with openness and non-judgmentalness (A) that leads to a significant shift in perspective, termed as reperceiving.” Shapiro et al. (2006) also highlighted four additional mechanisms: (1) self-regulation, (2) value clarification, (3) cognitive, emotional, and behavioral flexibility, and (4) exposure. Later, Tang and colleagues (Hözel et al., 2011; Tang et al., 2015; Tang, 2017) proposed that mindfulness practice includes at least three components that interact closely to enhanced self-regulation: enhanced attentional control, improved emotion regulation, and altered self-awareness. Different from other cognitive training, mindfulness-based training has pervasive effects: they not only promote young children’s social-emotional competence by reducing behavioral problems and increasing impulse control but also effectively enhance children’s cognitive abilities such as attention and inhibition (e.g., Crooks et al., 2020; Razza et al., 2020; Li-Grining et al., 2021).

A recent review on mindfulness-based intervention with young children suggested that over time, with practice and integration, mindfulness programs could support EF development in the early years (Bockmann and Yu, 2022). In addition, this review study also found noticeable variations in the structure, design, skills taught, frequency of practice, and duration of mindfulness-based interventions globally. These interventions all included breathwork and increasing awareness of sensations, feelings, and thoughts as their focus. Another review study has suggested a relatively specific rather than the general benefit of EF from mindfulness, with consistent improvement in inhibitory control and more variable advantages to working memory and cognitive shifting (Gallant, 2016). However, the above-mentioned interventions only employed parent or teacher reports and behavioral tasks to examine the effects of the mindfulness programs.

Neuroimaging techniques have been applied to identify the neural correlates and cognitive processes associated with mindful practices. Changes in cortical thickness (Lazar et al., 2005; Grant et al., 2010), gray-matter volume and/or density (Vestergaard-Poulsen et al., 2009; Hölzel et al., 2011), fractional anisotropy and axial and radial diffusivity (Tang et al., 2010, 2012) have been captured after mindfulness practices. Furthermore, research using fMRI has demonstrated that mindfulness practices increase performances on attentional control tasks (Tang et al., 2007), inhibitory control tasks (Jack et al., 2013), and working memory tasks (Mrazek et al., 2013). Despite the pervasive evidence from fMRI, this technique is limited in measuring vulnerable populations, such as those with trauma or young children under stress. The non-invasive fNIRS device can measure and monitor hemodynamic concentration changes in oxygenated (HbO) and deoxygenated (HbR) hemoglobin as an indicator of brain region activation. A recent study with a group of female participants impaired by stress or traumatic stress found that engagement in a 6-week mindfulness intervention was related to significant changes in performances in attentional control, emotional regulation, and working memory tasks and changes in activation in the frontopolar area, orbitofrontal cortex, and premotor cortex (Bergen-Cico et al., 2021). However, no neuroimaging evidence has been reported to prove the effectiveness of mindfulness-based interventions in preschoolers. To fill this gap, this study endeavors to explore the neural mechanisms of change by taking pre- and post-intervention fNIRS measurements of a group of 5- to 6-year-old children attending mindfulness-based interventions during their pre-school education. In addition, we aimed to explore whether fNIRS is an effective non-invasive means of measuring EF changes associated with mindfulness-based interventions. Specifically, the following hypotheses guided the current study:

H1: There is a significant change in young children’s cognitive shifting after participation in the mindfulness-based intervention.

H2: There is a significant change in young children’s inhibitory control after participation in the mindfulness-based intervention.

H3: There is a significant change in young children’s working memory after participation in the mindfulness-based intervention.

H4: The behavioral change in cognitive shifting is evidenced by fNIRS data.

H5: The behavioral change in inhibitory control is evidenced by fNIRS data.

H6: The behavioral change in working memory is evidenced by fNIRS data.

## Materials and methods

### Participants

Thirty-three preschoolers who attended the same upper class of the target preschool participated in this one-group pretest-posttest study, but two of the children failed to participate in the post examination and were excluded from the analysis. Shenzhen University’s Institutional Review Board approved all study procedures, and all participants’ parents gave consent for their children to participate in the study. The 31 participating children were right-handed and their months of age ranging from 62 to 73 months (*M* = 67.03, SD = 4.25). Among them, 19 were boys (*M* = 67.58, SD = 3.99, range 62–73 months) and 12 were girls (*M* = 66.17, SD = 4.28, range 62–73 months). There were no significant differences in age between the two groups (*t* = 0.91, *p* = 0.37). The intervention and data collection were conducted from October 2021 to January 2022. *Post hoc* power analysis using G*Power 3.1.9.7 showed that with a sample size of 31, α error probability, and power of 0.95, the effect size was 0.59, which is acceptably large.

### Measures

Three cognitive tasks were used to measure children’s executive function. The first task, DCCS, measures children’s cognitive shifting; the second task, missing scan, measures children’s working memory; and the third task, go/no-go, measures children’s inhibitory control. The three tasks were programmed using PsychToolBox (PTB) toolkit in Matlab. Stimuli were displayed on the computer screen, and responses were recorded by operating the keyboard. Before each task, participants were trained to make sure that they understood the rules of the tasks. During the test phases, the experimenter recorded each participant’s reaction time and responses, who clicked corresponding reactions on the keyboard. Children were instructed to look at the “+” on the screen during the rest phases and sit still.

#### Dimensional change card sort task

**FIGURE 1:**
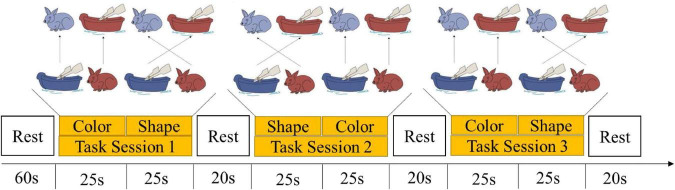
Experiment paradigm of the DCCS task.

The DCCS task has been used in the previous fNIRS studies to measure children’s cognitive shifting (Li et al., 2021b; Xie et al., 2021). A set of stimuli cards were displayed in the center of the screen. The stimuli card had two dimensions: shape and color. The target cards (a red boat and a blue rabbit) and test cards (e.g., a blue boat and a red rabbit) were matched in one dimension but did not match the other dimensions. There were three consecutive test sessions and four rest sessions in between. Each test session consisted of a pre and post-switch phase, with each phase lasting 25 s. The rules for matching were changed according to the experimenter’s instruction, and the rule order of the task was changed to avoid the learning effect (Figure 1): color→shape, shape→color, color→shape.

#### Go/no-go task

**FIGURE 2:**
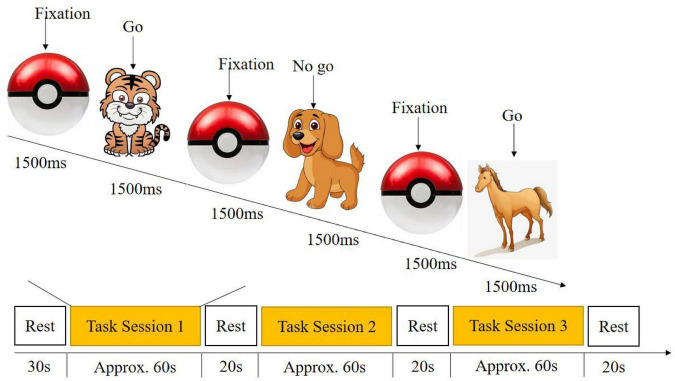
Experiment paradigm of the go/no-go task.

The go/no-go task was modified from Lahat et al.’s (2010) paradigm to measure children’s inhibitory control, as it has good validity and well-mapped neural bases (Wiebe et al., 2012). Children were asked to respond to the go stimulus (e.g., a cow, horse, or tiger) by pressing the space bar and not to respond to the no-go stimulus (e.g., dog). There were 4 go trials and 4 no-go trials in the training session, where children will be reminded of the rules should they respond incorrectly. Altogether, there were three task sessions, with 10 go trials and 10 no-go trials randomly distributed within each session (Figure 2).

#### Missing scan task

**FIGURE 3:**
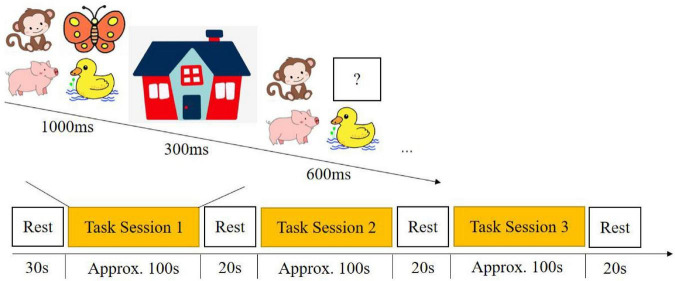
Test paradigm for the missing scan task.

The missing scan task was modified from Roman’s task to make it suitable for the fNIRS experiment paradigm, as it is suitable for measuring working memory capacity for 3- to 6- year-olds (Roman et al., 2014). A total of 30 animal figures were used as test stimuli. Examples of animals in the test set include monkey, pig, butterfly, and duck. Children were instructed to name pictures of each animal before carrying out the test to prevent the need to learn new vocabulary. The child used this label consistently and did not refer to another animal in the same set by the same name. Each time four animals appeared on the screen for 10 s, then disappeared into a “house” for 3 s, and then three animals re-appeared on the screen. Children were then instructed to call the name of the missing animal in 6 s before the next set of animals appeared on the screen. Each test session consisted of five trials, resulting in three test sessions and four rests (Figure 3).

#### The fNIRS examination

**FIGURE 4:**
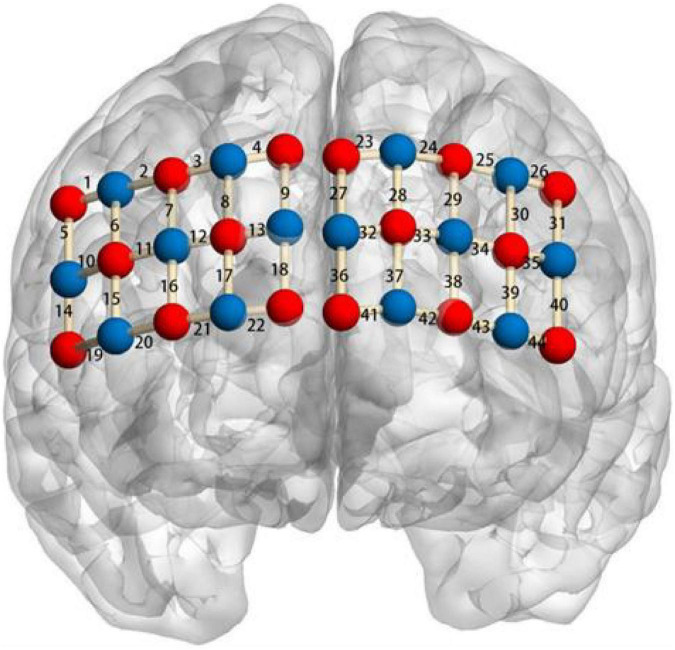
Localization of regions of interest. Right Ventrolateral prefrontal cortex (VLPFC): channel 16, 17, 21, 22; left VLPFC: channel 38, 39, 42, 43; right dorsolateral prefrontal cortex (DLPFC): channel 3, 4, 7, 8, 9, 12, 13; left DLPFC: channel 24, 25, 28, 29, 30, 33, 34; right posterior superior frontal cortex (PSFC): channel 1, 2, 5, 6; left PSFC: channel 26, 31; right temporal cortex (TC): channel 10, 11, 14, 15, 19, 20; left TC: channel 35, 40, 44; and Medial prefrontal cortex (MPFC): channel 18, 23, 27, 32, 36, 37, 41.

A multi-channel fNIRS system (Oxymon Mk III, Artinis, Netherlands) was used to collect the changes in oxygenated hemoglobin (HbO), deoxygenated hemoglobin (HbR), and total hemoglobin (HbT) when children performed the three executive function tasks. The optical intensity density values were corrected by the Beer–Lambert law and then converted into changes in the concentration of HbO and HbR. Following the study design of previous studies on young children’s EF(Schecklmann et al., 2010; Gu et al., 2017), a number of 30 optodes using a 3 × 10 light level stencil were located in the forehead, forming 44 fNIRS channels to cover the frontal area (see Figure 4). To ensure consistent light-level array positions for all participants, the lower middle of the array was positioned at the Fpz position, which is consistent with the 10–20 measurement system. Previous studies have shown that the frontal area was actively involved in executive function (Li et al., 2021a,b; Moriguchi, 2022). The sampling rate was set at 50Hz for data acquisition. A subject-specific differential pathlength factor (DPF) constant was calculated based on the age of each subject (Duncan et al., 1996): (DPF = 4.99 + 0.067 × Age^0.814^).

### Procedure

The 5-week mindfulness-based intervention design is conducted as follows. First, in the baseline pre-session, all participants completed the three executive functions, and their parents filled out questionnaires concerning their children’s demographic information. Print questionnaires and consent forms were enveloped and carried home by children to their parents, who gave consent and filled the questionnaires. Children then bring them back to the preschool, and class teachers collected these forms and passed them to the research team. Next, the participants were invited to complete the three executive function tasks in a quiet classroom at the preschool. Before the tasks, an experienced NIRS technician put the child-sized NIRS cap and installed optodes. At the same time, a student who majored in early childhood education or psychology engaged in story-book reading with the child. All three tasks were computerized using Psychophysics Toolbox extensions and displayed on a 55.35 cm × 31.13 cm Dell monitor. Children were trained to perform the tasks before each experiment began. For the DCCS and missing scan task, the experimenter recorded participants’ responses using the keyboard, and for the go/no-go task, children pressed on the space bar instead. Both responses and response time were recorded. After the baseline assessment, all the participants were engaged in a 5-week mindfulness training session per week. After the 5-week intervention, the participants were invited to complete the same executive function tasks (DCCS, missing scan, and go/no-go) while wearing fNIRS equipment.

The mindfulness training was adapted from Lv’s (2017) mindfulness training and Stewart and Braun’s (2017) mindfulness activities to make it both playful and mindful. The training package consisted of three parts that spanned ten sessions, two sessions a week, and 20 min per session. The first phase focused on breathwork and attention, which included activities such as introduction to mindful breathing, breathing like a frog, mountain raising, and rooted like a tree; the second phase focused on emotional awareness and regulation, which included activities such as mindful bubbles, fist squeeze, peaceful place, the power of blue, and joyful jellyfish; and the third phase focused on gratitude, which included activities such as loving-kindness, heart garden, animal dance, and floating smiles. A certified preschool mindfulness teacher and researcher in early childhood education and a researcher in mental health psychology adapted the training course. The class teacher of the participants received training from the certified teacher and delivered the mindfulness training during the school day.

### Data analysis

The participants’ behavioral results were exported from Matlab and calculated for the three experiment tasks. First, paired sample *t*-tests were conducted to examine whether there were significant differences in groups’ response time and correct rate before and after the mindfulness training. Furthermore, we grouped the participants by gender and explored whether the changes differed for boys and girls.

Next, for the blood oxygen concentration and deoxygenation concentration data of the 44 channels were first visually inspected to assess the quality of the signal. If the optical coupling between the optode and the scalp is not good, it will cause the whole channel to have high frequency signal interference coming from head movement, so such channels are removed before formal analysis (Brigadoi et al., 2014). Then, the NIRS-KIT software (Hou et al., 2021) was used to perform first-order baseline correction on the blood oxygen concentration and deoxygenation concentration data. Motion artifacts were removed using the DTTR algorithm (Fishburn et al., 2019). A bandpass filter (third-order Butterworth filter) with cut-off frequencies of 0.01–0.08 Hz (Pinti et al., 2019) was then applied to the data to reduce slow drifts and high-frequency noise.

After the fNIRS data were prepossessed, the HbO and HbR concentration were converted into *z*-scores using the mean value and *SD* of the HbO and HbR concentration changes during the rest phase, respectively. Next, a two-level mixed effect model Region of Interest (ROI) analysis was performed. At the first level analysis, GLM was performed for each channel and each subject by comparing the task to the rest phase. To increase the signal-to-noise ratio, the 44 channels were averaged into nine ROIs, where the time-series data were averaged within each ROI (Gu et al., 2017): the left ventrolateral prefrontal cortex (VLPFC), right VLPFC, left dorsolateral prefrontal cortex (DLPFC), right DLPFC, left posterior superior frontal cortex (PSFC), right PSFC, left temporal cortex (TC), right TC, and medial prefrontal cortex (MPFC). At the second level group analysis, the pre- and post-intervention betas for each ROI were compared using paired sample *t*-test by group level of the total sample, and the *p* values were FDR adjusted. We also explored whether boys showed different patterns from girls by grouping the total sample by gender.

## Results

### Behavioral results

**FIGURE 5:**
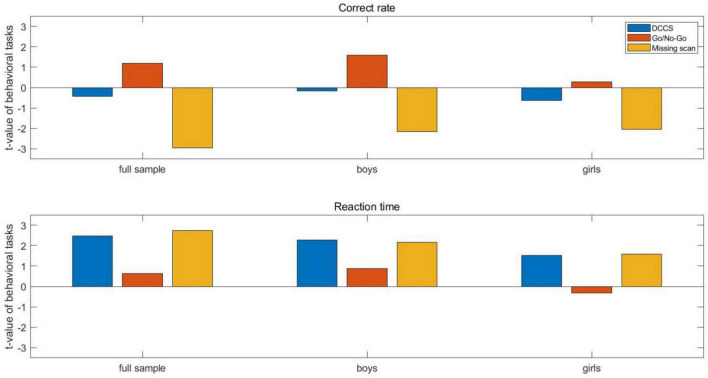
Bar chart for the behavioral results of the three tasks before and after intervention for the full sample, boys’ group, and girls’ group, respectively.

Paired sample *t*-test revealed that two out of the three tasks showed significant improvement after mindfulness-based intervention (Table 1 and Figure 5). First, for the DCCS task, children’s response time shortened, showing improved cognitive shifting abilities and supporting H1. Second, children’s correct rate improved, and response time shortened for the missing scan task, showing improved working memory abilities and supporting H3. Finally, for the go/no-go task, there was no significant change in children’s correct rate or response time, failing to support H2. We further explored whether boys and girls were different in the behavioral changes before and after the mindfulness-based intervention by doing paired sample *t*-tests for boys’ and girls’ groups separately. The results show no significant differences in girls’ behavioral results before and after the intervention. Still, there were significant differences in boys’ behavioral results: reaction time for the DCCS and missing scan task shortened, and the correct rate for the missing scan task improved. When comparing the performances between boys and girls, there were no significant differences in pre- and post-interventions for the three tasks (*p*s > 0.05), except for the reaction time in the go/no-go task in post-intervention (*t* = –2.56, *p* < 0.05).

**TABLE 1:** Results for paired sample *t*-test of behavioral tasks before and after the intervention.

Tasks		Correct rate			Reaction time		
			
		*M* (*SD*)	*t*-value	*P*-value	*M (SD)*	*t*-value	*P*-value
**Total sample (*N* = 31)**							
DCCS	Pre	0.93(0.07)	–0.42	0.67	5.6(0.88)	**2.48**	0.02*
	Post	0.94(0.06)			5.3(0.66)		
Go/No-Go	Pre	0.94(0.06)	1.19	0.24	1.14(0.09)	0.62	0.54
	Post	0.92(0.06)			1.13(0.10)		
Missing Scan	Pre	0.44(0.18)	**–2.96**	0.01**	5.06(0.51)	**2.73**	0.01**
	Post	0.51(0.19)			4.82(0.51)		
**Girls (*N* = 12)**							
DCCS	Pre	0.94(0.05)	–0.62	0.55	5.88(1.01)	1.52	0.16
	Post	0.95(0.04)			5.48(0.72)		
Go/No-Go	Pre	0.94(0.06)	0.28	0.78	1.18(0.06)	–0.33	0.75
	Post	0.93(0.07)			1.18(0.07)		
Missing Scan	Pre	0.38(0.18)	–2.05	0.07	5.14(0.46)	1.58	0.14
	Post	0.49(0.17)			4.93(0.42)		
**Boys (*N* = 19)**							
DCCS	Pre	0.92(0.08)	–0.17	0.87	5.40(0.75)	**2.27**	0.04*
	Post	0.92(0.07)			5.19(0.61)		
Go/No-Go	Pre	0.94(0.05)	1.59	0.13	1.12(0.09)	0.87	0.40
	Post	0.92(0.06)			1.10(0.10)		
Missing Scan	Pre	0.47(0.18)	**–2.16**	0.04*	5.00(0.54)	**2.17**	0.04*
	Post	0.53(0.20)			4.76(0.56)		

**p < 0.01; *p < 0.05. Bold values indicate significant values.

### fNIRS results

First, a set of two-sample (independent groups) *t*-tests was conducted to determine any significant difference in the mean HbO and HbR increase before and after the mindfulness-based intervention. As multiple channels were involved, all the results were corrected for multiple comparisons using the false discovery rate (FDR), and the adjusted significance level of the p-value was set at 0.05. The results indicated a significant between-group difference in the right DLPFC. As shown in Tables 2, 3, a significant increase in HbO (*t* = –3.13, *p* < 0.05) and a significant decrease in HbR (*t* = 3.05, *p* < 0.05) was observed in the right DLPFC after the intervention, supporting H6. However, H4 and H5 were not supported.

**TABLE 2:** Comparison of increases in HbO before and after the intervention (full sample).

Task	ROI	Pre-intervention *M* (SD)	Post-intervention *M* (SD)	*t*-value	*P*-value
DCCS	Left VLPFC	–0.98(1.79)	–0.25(1.81)	–1.69	0.45
	Right VLPFC	–0.61(1.62)	–0.19(1.43)	–1.06	0.54
	Left DLPFC	–0.50(1.20)	–0.21(1.70)	–0.88	0.56
	Right DLPFC	–0.84(1.77)	0.38(1.40)	–**3.13**	0.03*
	Left PSFC	–0.40(1.13)	–0.81(1.98)	0.73	0.56
	Right PSFC	–0.36(1.25)	0.13(1.19)	–1.30	0.51
	Right TC	–0.23(1.09)	–0.34(2.31)	0.25	0.80
	Left TC	0.00(1.46)	0.37(2.23)	–0.68	0.56
	MFPC	–1.21(1.55)	–0.67(1.78)	–1.35	0.51
Go/No-Go	Left VLPFC	–0.15(1.12)	–0.41(1.50)	0.63	0.80
	Right VLPFC	0.06(1.27)	0.22(1.41)	–0.39	0.90
	Left DLPFC	0.14(1.14)	–0.20(1.99)	0.81	0.80
	Right DLPFC	–0.20(1.24)	0.02(1.36)	–0.85	0.80
	Left PSFC	–0.09(0.62)	0.02(2.86)	–0.12	0.91
	Right PSFC	0.28(1.25)	–0.24(1.90)	0.67	0.80
	Right TC	0.02(0.97)	–0.48(1.74)	1.28	0.80
	Left TC	–0.14(1.51)	–0.56(1.54)	1.15	0.80
	MFPC	0.17(1.16)	0.09(1.63)	0.21	0.91
Missing Scan	Left VLPFC	–0.32(1.04)	–0.03(1.48)	–0.91	0.56
	Right VLPFC	–0.49(1.36)	0.08(1.06)	–1.81	0.36
	Left DLPFC	0.12(1.46)	0.04(1.12)	0.24	0.81
	Right DLPFC	–0.12(1.67)	0.48(1.49)	–1.83	0.36
	Left PSFC	0.12(1.31)	–1.04(1.84)	1.58	0.43
	Right PSFC	0.60(1.17)	0.10(0.90)	1.33	0.43
	Right TC	0.11(1.02)	0.30(1.62)	–0.58	0.66
	Left TC	–0.11(0.86)	0.18(1.17)	–1.19	0.43
	MFPC	–0.41(0.79)	–0.25(1.32)	–0.56	0.66

*p < 0.05. Bold values indicate significant values.

**TABLE 3:** Comparison of increases in HbR before and after the intervention (full sample).

Task	ROI	Pre-intervention *M* (SD)	Post-intervention *M* (SD)	*t*-value	*P*-value
DCCS	Left VLPFC	–0.05(1.49)	–0.35(1.65)	0.69	0.60
	Right VLPFC	–0.03(1.41)	–0.63(1.80)	1.54	0.37
	Left DLPFC	–0.16(1.02)	–0.62(1.32)	1.42	0.37
	Right DLPFC	0.34(1.44)	–0.42(1.35)	**3.05**	0.04*
	Left PSFC	–0.51(0.74)	–0.72(2.12)	0.30	0.77
	Right PSFC	–1.85(2.51)	–0.41(1.45)	–1.86	0.37
	Right TC	–0.38(1.58)	–0.64(1.88)	0.63	0.60
	Left TC	–0.08(1.54)	–0.35(1.37)	0.80	0.60
	MFPC	–0.01(1.46)	–0.26(1.48)	0.78	0.60
Go/No-Go	Left VLPFC	0.06(1.51)	–0.33(1.30)	1.10	0.58
	Right VLPFC	–0.28(1.29)	–0.51(1.61)	0.62	0.58
	Left DLPFC	–0.18(0.86)	0.16(1.27)	–1.13	0.58
	Right DLPFC	–0.18(1.04)	0.03(1.39)	–0.65	0.58
	Left PSFC	–0.22(1.28)	0.25(1.12)	–0.99	0.58
	Right PSFC	–0.11(1.98)	–1.75(2.45)	1.32	0.58
	Right TC	–0.22(1.19)	–0.03(1.76)	–0.56	0.58
	Left TC	–0.65(1.49)	–0.21(1.18)	–1.17	0.58
	MFPC	–0.25(1.08)	0.00(1.32)	–0.79	0.58
Missing Scan	Left VLPFC	0.11(1.01)	0.20(0.94)	–0.36	0.86
	Right VLPFC	0.05(1.18)	0.01(0.98)	0.13	0.90
	Left DLPFC	0.09(0.97)	–0.27(1.00)	1.27	0.64
	Right DLPFC	0.06(1.22)	–0.39(0.97)	1.78	0.43
	Left PSFC	0.11(0.90)	–0.30(1.02)	0.92	0.69
	Right PSFC	–0.27(0.93)	0.10(1.06)	–0.77	0.69
	Right TC	–0.15(0.98)	0.09(1.27)	–0.86	0.69
	Left TC	0.29(1.05)	–0.22(1.42)	1.72	0.43
	MFPC	0.08(0.94)	0.00(0.88)	0.30	0.86

*p < 0.05. Bold values indicate significant values.

We also explored whether the changes in neural activation before and after the mindful-based intervention differed for boys and girls. Therefore, paired sample *t*-tests of the pre- and post-intervention brain activations were conducted for girls and boys separately. Table 4 showed that for girls, there were no significant changes in HbO before and after the mindful based intervention (*p*s > 0), but Table 5 showed significant increase in HbR in MPFC (*t* = –3.95, *p* < 0.05) and decrease in right VLPFC (*t* = 3.99, *p* < 0.05) and right DLPFC (*t* = 4.48, *p* < 0.05). Table 6 showed that for boys, there were no significant changes in HbO before and after the mindful-based intervention (*p*s > 0), but Table 7 showed a significant decrease in HbR in the right DLPFC (*t* = 3.56, *p* < 0.05).

**TABLE 4:** Comparison of increases in HbO before and after the intervention for girls (*N* = 12).

Task	ROI	Pre-intervention *M* (*SD*)	Post-intervention *M* (*SD*)	*t*-value	*P*-value
DCCS	Left VLPFC	–1.26(1.78)	–0.52(2.01)	–0.98	0.79
	Right VLPFC	–1.20(2.11)	–0.19(1.43)	–1.35	0.79
	Left DLPFC	–0.55(1.75)	–0.91(1.56)	0.62	0.99
	Right DLPFC	–1.09(2.12)	0.22(1.45)	–1.65	0.79
	Left PSFC	–0.74(1.71)	–0.66(1.90)	–0.14	0.99
	Right PSFC	0.00(2.75)	–0.52(2.31)	1.65	0.79
	Right TC	–0.79(1.07)	–0.53(3.22)	–0.28	0.99
	Left TC	0.11(2.13)	0.38(3.31)	–0.21	0.99
	MFPC	–1.55(1.73)	–1.55(1.68)	0.01	0.99
Go/No-Go	Left VLPFC	–0.20(0.87)	0.14(0.98)	–0.74	0.89
	Right VLPFC	–0.23(1.33)	0.34(1.70)	–0.71	0.89
	Left DLPFC	0.30(0.87)	0.34(1.93)	–0.06	0.98
	Right DLPFC	–0.12(1.16)	–0.10(1.40)	–0.03	0.98
	Left PSFC	0.22(0.64)	–0.16(0.35)	1.60	0.82
	Right PSFC	–0.43(0.78)	0.83(1.60)	–2.18	0.82
	Right TC	–0.24(1.09)	–0.54(1.09)	0.52	0.92
	Left TC	0.00(1.36)	–0.66(1.04)	1.23	0.82
	MFPC	0.14(1.04)	0.32(1.63)	–0.32	0.97
Missing Scan	Left VLPFC	–0.44(0.94)	–0.11(1.90)	–0.50	0.81
	Right VLPFC	–0.32(1.51)	0.07(0.65)	–0.70	0.81
	Left DLPFC	–0.29(1.25)	0.37(0.72)	–2.05	0.29
	Right DLPFC	–0.42(1.13)	0.41(0.77)	–2.16	0.29
	Left PSFC	–0.40(1.44)	–0.22(1.10)	–0.27	0.87
	Right PSFC	0.84(1.41)	0.23(0.38)	0.84	0.81
	Right TC	0.24(1.14)	0.17(0.99)	0.17	0.87
	Left TC	–0.21(0.99)	0.05(1.08)	–0.63	0.81
	MFPC	–0.43(0.74)	–0.04(1.50)	–0.71	0.81

**TABLE 5:** Comparison of increases in HbR before and after the intervention for girls (*N* = 12).

Task	ROI	Pre-intervention *M* (*SD*)	Post-intervention *M* (*SD*)	*t*-value	*P*-value
DCCS	Left VLPFC	0.19(1.58)	–0.19(1.17)	0.60	0.72
	Right VLPFC	0.10(1.78)	–0.26(1.04)	0.69	0.72
	Left DLPFC	–0.06(0.73)	–0.88(1.32)	1.68	0.72
	Right DLPFC	0.41(1.81)	–0.04(1.53)	0.93	0.72
	Left PSFC	–0.02(0.49)	–0.30(0.36)	0.57	0.72
	Right PSFC	–4.41(3.75)	–1.04(1.56)	–2.18	0.72
	Right TC	–0.16(1.45)	–0.49(2.34)	0.46	0.72
	Left TC	0.42(1.80)	0.18(1.40)	0.42	0.72
	MFPC	–0.18(1.75)	0.01(0.83)	–0.37	0.72
Go/No-Go	Left VLPFC	–0.11(1.24)	0.21(1.05)	–0.85	0.46
	Right VLPFC	–0.71(1.06)	0.21(1.25)	–2.09	0.15
	Left DLPFC	–0.20(0.85)	0.27(1.24)	–0.96	0.46
	Right DLPFC	–0.60(1.01)	0.49(1.45)	–2.05	0.15
	Left PSFC	–1.00(1.44)	–0.46(0.69)	–1.22	0.46
	Right PSFC	–0.65(2.11)	–1.43(3.85)	0.19	0.88
	Right TC	–0.43(1.46)	0.68(2.16)	–2.17	0.15
	Left TC	–1.02(2.15)	0.00(1.01)	–1.29	0.40
	MFPC	–0.54(0.59)	0.45(1.10)	–**3.95**	0.02*
Missing Scan	Left VLPFC	0.57(1.12)	–0.19(1.02)	1.96	0.14
	Right VLPFC	0.44(0.74)	–0.40(0.61)	**3.99**	0.01*
	Left DLPFC	0.25(1.10)	–0.60(0.78)	2.14	0.13
	Right DLPFC	0.54(1.00)	–0.65(0.73)	**4.48**	0.01*
	Left PSFC	0.24(1.02)	–0.34(1.16)	0.54	0.72
	Right PSFC	–0.85(1.99)	–0.49(0.12)	–0.24	0.85
	Right TC	–0.53(1.12)	–0.17(0.70)	–1.15	0.36
	Left TC	0.57(1.22)	–0.17(0.89)	1.50	0.24
	MFPC	0.34(0.79)	–0.25(0.68)	2.14	0.13

*p < 0.05. Bold values indicate significant values.

**TABLE 6:** Comparison of increases in HbO before and after the intervention for boys (*N* = 19).

Task	ROI	Pre-intervention *M* (SD)	Post-intervention *M* (SD)	*t*-value	*P*-value
DCCS	Left VLPFC	–0.79(1.82)	–0.08(1.71)	–1.36	0.34
	Right VLPFC	–0.24(1.13)	–0.18(1.46)	–0.12	0.90
	Left DLPFC	–0.46(0.72)	0.24(1.67)	–1.88	0.23
	Right DLPFC	–0.67(1.55)	0.48(1.40)	–2.84	0.10
	Left PSFC	–0.27(0.97)	–0.86(2.13)	0.79	0.54
	Right PSFC	–0.45(0.94)	0.29(0.96)	–1.76	0.28
	Right TC	0.09(0.98)	–0.23(1.68)	0.72	0.54
	Left TC	–0.06(0.89)	0.36(1.27)	–1.16	0.39
	MFPC	–1.00(1.44)	–0.11(1.64)	–1.97	0.23
Go/No-Go	Left VLPFC	–0.12(1.28)	–0.76(1.68)	1.07	0.59
	Right VLPFC	0.24(1.24)	0.15(1.24)	0.20	0.84
	Left DLPFC	0.03(1.29)	–0.54(2.01)	1.13	0.59
	Right DLPFC	–0.25(1.32)	0.09(1.37)	–1.11	0.59
	Left PSFC	–0.20(0.62)	0.09(3.41)	–0.23	0.84
	Right PSFC	0.46(1.32)	–0.51(1.96)	1.05	0.59
	Right TC	0.17(0.89)	–0.45(2.05)	1.16	0.59
	Left TC	–0.22(1.63)	–0.50(1.82)	0.54	0.82
	MFPC	0.18(1.25)	–0.05(1.66)	0.48	0.82
Missing Scan	Left VLPFC	–0.25(1.12)	0.02(1.21)	–0.79	0.53
	Right VLPFC	–0.59(1.28)	0.08(1.28)	–1.80	0.53
	Left DLPFC	0.38(1.56)	–0.17(1.29)	1.19	0.53
	Right DLPFC	0.07(1.94)	0.53(1.82)	–0.95	0.53
	Left PSFC	0.32(1.31)	–1.35(2.03)	1.76	0.53
	Right PSFC	0.54(1.20)	0.07(1.01)	1.03	0.53
	Right TC	0.04(0.97)	0.37(1.92)	–0.74	0.53
	Left TC	–0.05(0.79)	0.26(1.25)	–1.01	0.53
	MFPC	–0.40(0.84)	–0.39(1.22)	–0.03	0.97

**TABLE 7:** Comparison of increases in HbR before and after the intervention for boys (*N* = 19).

Task	ROI	Pre-intervention *M* (SD)	Post-intervention *M* (SD)	*t*-value	*P*-value
DCCS	Left VLPFC	–0.21(1.45)	–0.45(1.92)	0.41	0.77
	Right VLPFC	–0.11(1.17)	–0.86(2.14)	1.36	0.66
	Left DLPFC	–0.22(1.17)	–0.45(1.33)	0.54	0.77
	Right DLPFC	0.30(1.21)	–0.66(1.20)	**3.56**	0.02*
	Left PSFC	–0.69(0.76)	–0.89(2.50)	0.20	0.85
	Right PSFC	–1.21(1.93)	–0.25(1.49)	–1.14	0.66
	Right TC	–0.51(1.68)	–0.72(1.62)	0.42	0.77
	Left TC	–0.39(1.30)	–0.68(1.29)	0.68	0.77
	MFPC	0.10(1.29)	–0.43(1.78)	1.25	0.66
Go/No-Go	Left VLPFC	0.17(1.68)	–0.66(1.36)	1.67	0.51
	Right VLPFC	0.00(1.38)	–0.96(1.68)	2.03	0.51
	Left DLPFC	–0.17(0.89)	0.09(1.31)	–0.66	0.66
	Right DLPFC	0.09(0.99)	–0.26(1.31)	0.96	0.66
	Left PSFC	0.07(1.18)	0.51(1.17)	–0.69	0.66
	Right PSFC	0.03(2.08)	–1.83(2.36)	1.38	0.63
	Right TC	–0.09(1.02)	–0.44(1.39)	0.89	0.66
	Left TC	–0.41(0.86)	–0.35(1.28)	–0.18	0.86
	MFPC	–0.07(1.28)	–0.28(1.39)	0.46	0.73
Missing Scan	Left VLPFC	–0.18(0.85)	0.45(0.81)	–2.29	0.31
	Right VLPFC	–0.20(1.35)	0.27(1.09)	–1.13	0.79
	Left DLPFC	–0.02(0.89)	–0.06(1.09)	0.11	0.93
	Right DLPFC	–0.25(1.27)	–0.22(1.08)	–0.09	0.93
	Left PSFC	0.06(0.93)	–0.29(1.06)	0.68	0.79
	Right PSFC	–0.12(0.65)	0.24(1.14)	–0.69	0.79
	Right TC	0.08(0.84)	0.24(1.51)	–0.40	0.89
	Left TC	0.12(0.92)	–0.24(1.70)	0.97	0.79
	MFPC	–0.08(1.00)	0.16(0.96)	–0.65	0.79

*p < 0.05. Bold values indicate significant values.

The observed changes in the HbO and HbR concertation in the nine ROIs during the three tasks for the full sample, girls’ group, and boys’ group are averaged within group for each ROI and are shown in Supplementary Figures 1–6, respectively.

## Discussion

First, this study found that after the mindfulness-based intervention, children’s behavioral performance significantly improved in the DCCS task, indicating that the mindfulness-based intervention effectively enhanced children’s behavioral scores in the cognitive shifting. This finding is consistent with previous studies, confirming a positive effect of mindfulness training on children’s cognitive shifting (Flook et al., 2015; Bockmann and Yu, 2022). Second, this study found that children improved their behavioral performance in the Missing Scan task after the intervention, indicating that the mindfulness training was also effective in increasing children’s working memory span, which is consistent with previous studies (Janz et al., 2019; Razza et al., 2020). Third, this study did not find improvements in children’s behavioral performance in the Go-No-Go task (even lower scores in post-intervention), indicating no significant changes in children’s inhibition control after the mindfulness training, which failed to provide supplementary evidence to the existing literature (Flook et al., 2015). The reasons for this non-significant change may be that the current Go-No-Go task was designed as having the average number of the go and no-go trials, which limited the opportunities of the children to perform in the no-go trials, as the no-go trials were generally related to higher mindfulness (Logemann-Molnár et al., 2022). Therefore, future studies may enlarge the number of no-go trails to increase the opportunities for mindfulness-related sessions/events for the children to react. Generally speaking, mindfulness training is effective in enhancing young children’s EF, which corroborates with the findings of a recent literature review. However, the longer duration and higher training frequency tend to improve (Bockmann and Yu, 2022). It was also interesting to find that when separating the boys and girls, changes in the behavioral tasks (DCCS and missing scan) were significant only in the boys’ group, indicating that the mindfulness-based intervention benefited boys more than girls. This finding corroborates with the existing literature in which boys initially showed more somatic complaints than girls did and this difference disappeared by the end of the mindfulness-based intervention (Semple et al., 2010). Furthermore, the ages differences (despite non-significant) might explain for the different results for gender groups, which deserves further investigation.

Second, this study found a significant increase in HbO activation and a significant decrease in HbR activation in the right DLPFC after the mindfulness-based intervention in the total sample and the boy’s group, which indicated that fNIRS data also evidenced the behavioral changes in cognitive shifting. This finding is congruent with previous studies, which suggest mindfulness practices stabilize attention and improve cognitive flexibility (Bulzacka et al., 2017; Vieth and von Stockhausen, 2022). An individual engaging in the early stage of mindfulness practice often utilizes the DLPFC and parietal cortex (Posner et al., 2015; Tang et al., 2015) to try to get into the mediative state, which supports the findings of the current study with a group of preschoolers who were new to the mindfulness practice. Using the IAA model (Shapiro et al., 2006) and the attention regulation as components of the mindfulness mechanism (Tang et al., 2015), they both consider mindfulness practice to improve attentional processes by improving sustained attention and better monitoring as well as effective shifting between task set (i.e., cognitive shifting; Vieth and von Stockhausen, 2022).

Third, this study found a significant decrease in HbR activation in the MPFC after mindfulness-based intervention in the girl’s group, despite non-significant changes in go/no-go behavioral results. This indicated that changes in girls’ brain activation during inhibitory control tasks were not reflected in the behavioral performance. This is somewhat incongruent with a review study that found all but one of the studies reported mindfulness practice-related improvements to the inhibition outcomes (Gallant, 2016). Using the above-mentioned frameworks (Shapiro et al., 2006; Tang et al., 2015), mindfulness practices contribute to inhibitory control by maintaining attentional focus on a task (Vieth and von Stockhausen, 2022).

Finally, the study found a significant decrease in HbR activation in the right VLPFC and right DLPFC after mindfulness-based intervention in the girl’s group during the working memory task, but not for the boy’s group or the total sample. Despite incongruence between behavioral performance and neural activations, the findings jointly highlight that mindfulness-based intervention was beneficial for preschoolers’ working memory, which is congruent with previous studies (Jha et al., 2010; Mrazek et al., 2013). Jha et al. (2010) revealed that mindfulness training helped people reduce their stress levels, facilitating them to perform better in working memory tasks. This finding implied that mindfulness training might reduce the brain burden caused by stress during the working memory task to reduce brain activations. Coincidently, Mrazek et al. (2013) found that mindfulness training reduced mind wandering during the working memory task. This may be one reason for the decreased brain activation during the working memory task after mindfulness training, as there would be less burden caused by distracting thoughts. Furthermore, the neural efficiency model also postulates that in medium- to low-difficulty cognitive tasks, high performers tend to show lower brain activation than low performers due to higher efficiency in allocating neural resources (Dunst et al., 2014), which is also found in bilingual young children with advanced bilinguals showing less brain activation than less-advanced bilinguals (Xie et al., 2021). However, some sleep studies also found that decreased brain activation during a working memory task was associated with sleep-deprivation vulnerability (Mu et al., 2005). Therefore, the mechanism of this activation decreased during the working memory after mindfulness training deserves further investigation. Nonetheless, the current study suggests a relatively specific rather than general benefit of a mindfulness-based intervention to the three components of preschoolers’ EF, highlighting the advantages of examining all three components of EF simultaneously.

## Limitations and future research

The limitations of the current study are worth mentioning. First, given the wide variations of mindfulness-based interventions in the current literature, the existing study used two sessions per week, 5 weeks in total, which might not be enough duration and frequency for enhancing young children’s EF. This might lead to different patterns of change between behavioral performance and brain activations and between boys and girls. Second, all the participants were in the intervention group. Without a control group, the intervention effect is not convincing enough. Taking the limitations mentioned above, future research shall consider mindfulness training with a longer duration and a higher frequency. If possible, groups with different frequencies, duration, and gender might provide more evidence of the benefit of duration and frequency of the training. Furthermore, future studies shall consider intervention versus control group design to substantiate the benefits of mindfulness training.

## Conclusion and implications

The current study found that a 5-week, twice-per-week mindfulness training can enhance young children’s cognitive shifting and working memory, especially for boys. These changes in behavioral tasks were evidenced by significant changes in brain activation during cognitive shifting and working memory tasks. Furthermore, girls’ brain activation was significantly different during the missing scan task despite not being revealed in the behavioral results. The current study provided preliminary evidence that preschoolers can benefit from mindfulness training and implies that integrating it into the preschool curriculum might have some training effects. Furthermore, it implies that fNIRS is an effective tool in detecting neural activations changes brought by mindfulness training in young children.

## Data availability statement

The raw data supporting the conclusions of this article will be made available by the authors without undue reservation.

## Ethics statement

The studies involving human participants were reviewed and approved by The study was conducted in accordance with the Declaration of Helsinki, and the protocol was approved by the Research Ethics committee of Shenzhen University (PN-2021-038, approval date October 14, 2021). Written informed consent to participate in this study was provided by the participants’ legal guardian/next of kin.

## Author contributions

SX contributed to project conceptualization, data collection, and original manuscript drafting. CG and JL contributed to data collection, processing, analysis, and statistical analysis. HL constructive discussions and manuscript revision. DW contributed to constructive discussions and manuscript drafting. XC contributed to project conceptualization and research design. CC contributed to data processing, analysis, manuscript revision, and supervision. All authors contributed to the article and approved the submitted version.
