# Supplementary material for: Enhancing Chinese preschoolers’ executive function via mindfulness training: An fNIRS study
Source: Front Behav Neurosci. 2022 Aug 25;16:961797. doi: 10.3389/fnbeh.2022.961797 (PMC9452775; doi:10.3389/fnbeh.2022.961797)
Supplement: Supplementary file 1 [file Data_Sheet_1.PDF]

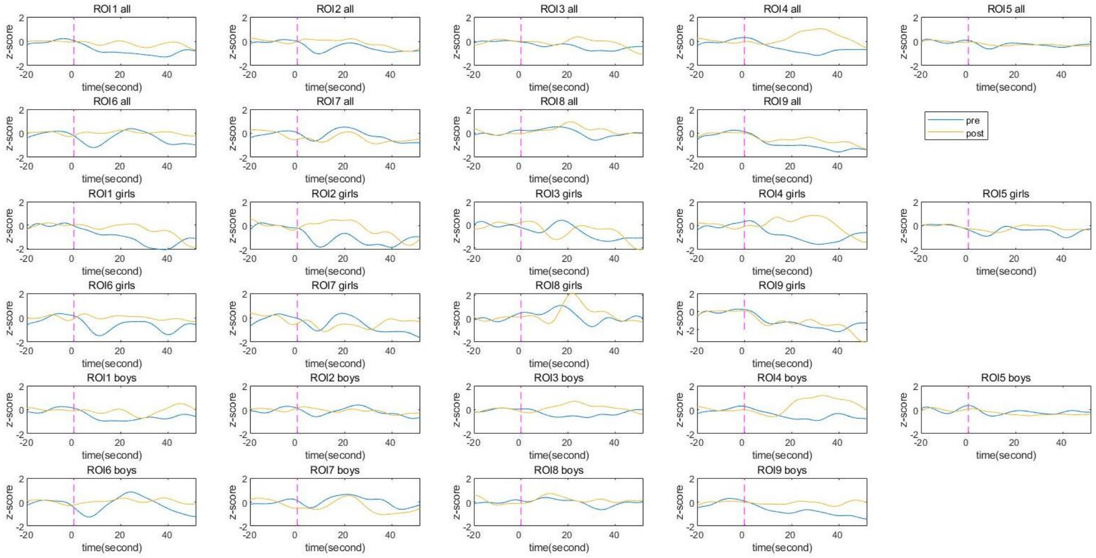

Supplementary Figure 1. The observed changes in the HbO concertation in the nine ROIs during the DCCS task. The blue and yellow lines are for pre-intervention and post-intervention, respectively. ROI 1 = left VLPFC; ROI 2 = right VLPFC; ROI 3 = left DLPFC; ROI 4= right DLPFC; ROI 5 = left PSFC; ROI 6 = right PSFC; ROI 7 = right TC; ROI 8 =left TC; ROI 9 =MFPC.

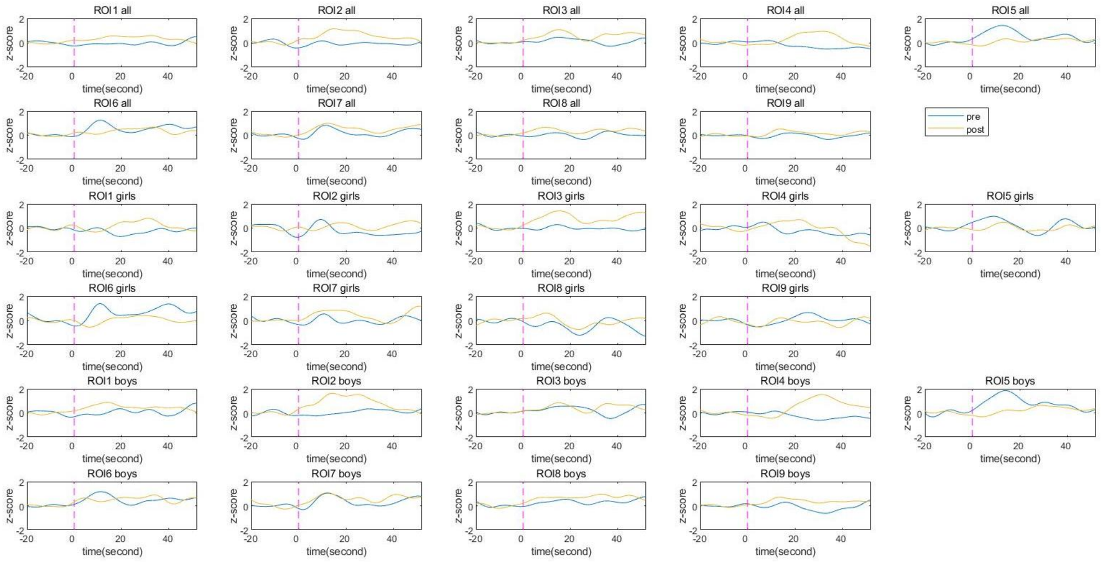

Supplementary Figure 2. The observed changes in the HbR concertation in the nine ROIs during the DCCS task. The blue and yellow lines are for pre-intervention and post-intervention, respectively. ROI 1 = left VLPFC; ROI 2 = right VLPFC; ROI 3 = left DLPFC; ROI 4= right DLPFC; ROI 5 = left PSFC; ROI 6 = right PSFC; ROI 7 = right TC; ROI 8 =left TC; ROI 9 =MFPC.

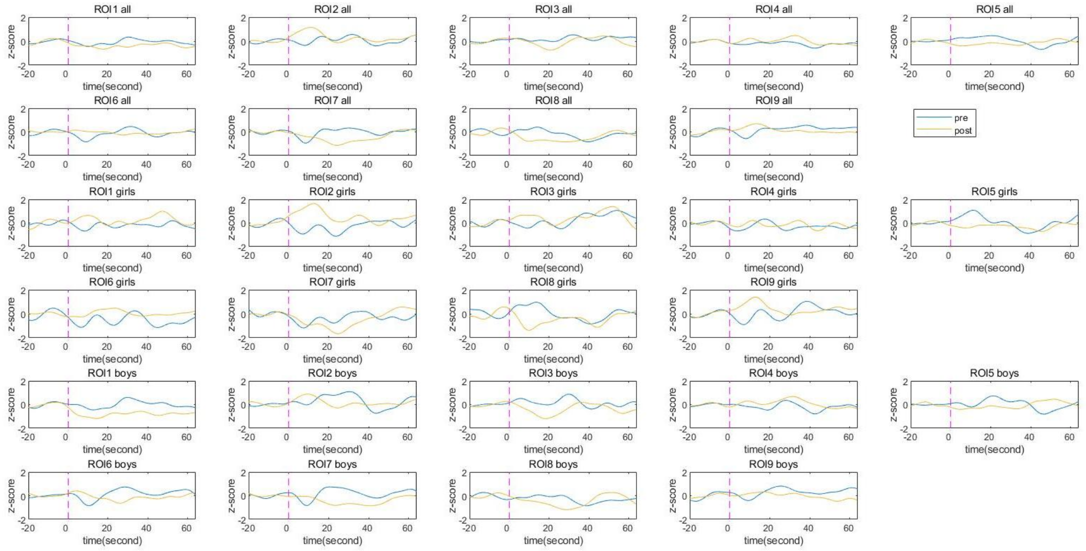

Supplementary Figure 3. The observed changes in the HbO concertation in the nine ROIs during the go/no-go task. The blue and yellow lines are for pre-intervention and post-intervention, respectively. ROI 1 = left VLPFC; ROI 2 = right VLPFC; ROI 3 = left DLPFC; ROI 4= right DLPFC; ROI 5 = left PSFC; ROI 6 = right PSFC; ROI 7 = right TC; ROI 8 =left TC; ROI 9 =MFPC.

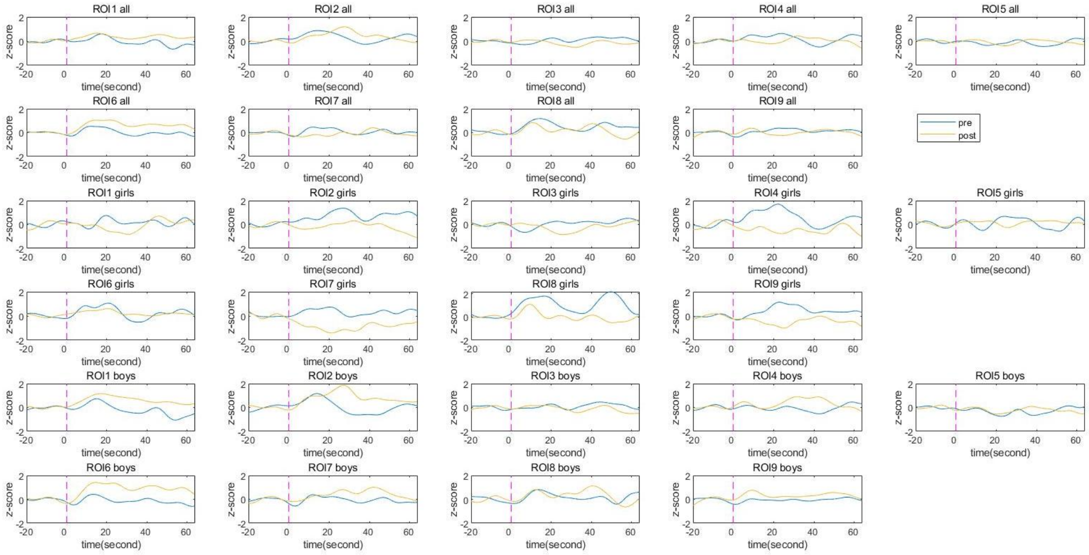

Supplementary Figure 4. The observed changes in the HbR concertation in the nine ROIs during the go/no-go task. The blue and yellow lines are for pre-intervention and post-intervention, respectively. ROI 1 = left VLPFC; ROI 2 = right VLPFC; ROI 3 = left DLPFC; ROI 4= right DLPFC; ROI 5 = left PSFC; ROI 6 = right PSFC; ROI 7 = right TC; ROI 8 =left TC; ROI 9 =MFPC.

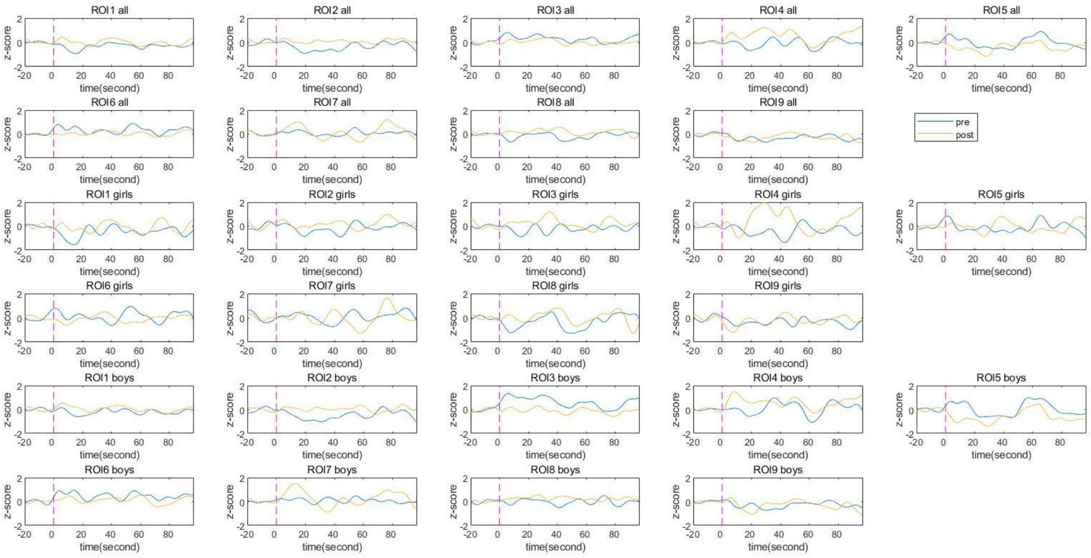

Supplementary Figure 5. The observed changes in the HbO concertation in the nine ROIs during the missing scan task. The blue and yellow lines are for pre-intervention and post-intervention, respectively. ROI 1 = left VLPFC; ROI 2 = right VLPFC; ROI 3 = left DLPFC; ROI 4= right DLPFC; ROI 5 = left PSFC; ROI 6 = right PSFC; ROI 7 = right TC; R OI 8 =left TC; ROI 9 =MFPC.

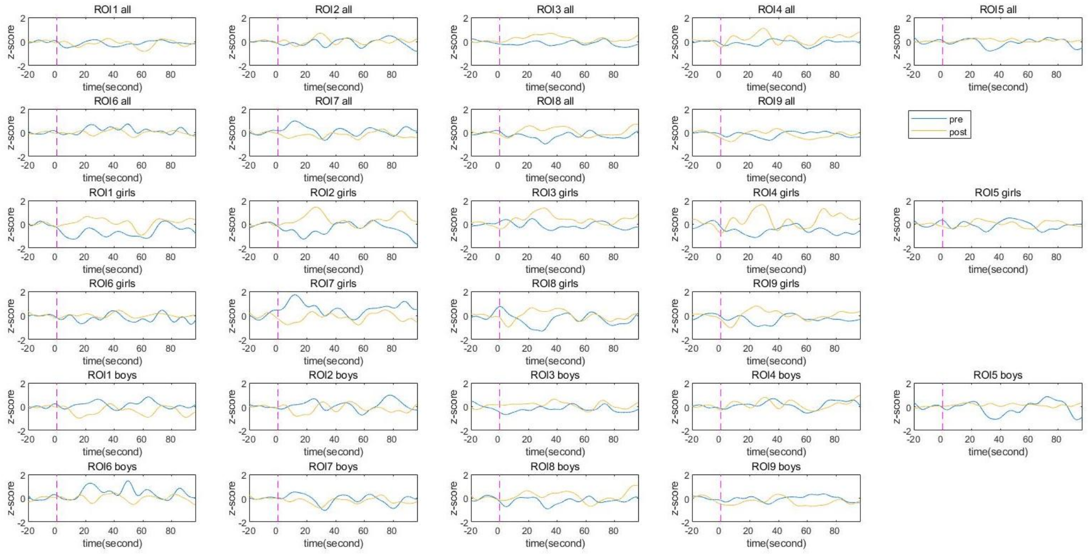

Supplementary Figure6. The observed changes in the HbR concertation in the nine ROIs during the missing scan task. The blue and yellow lines are for pre-intervention and post-intervention, respectively. ROI 1 = left VLPFC; ROI 2 = right VLPFC; ROI 3 = left DLPFC; ROI 4= right DLPFC; ROI 5 = left PSFC; ROI 6 = right PSFC; ROI 7 = right TC; ROI 8 =left TC; ROI 9 =MFPC.
